# Analyzing Disparity and Rates of Morphological Evolution with Model-Based Phylogenetic Comparative Methods

**DOI:** 10.1093/sysbio/syab079

**Published:** 2021-12-02

**Authors:** Thomas F Hansen, Geir H Bolstad, Masahito Tsuboi

**Affiliations:** Department of Biology, CEES & Evogene, University of Oslo, Oslo, Norway; Norwegian Institute for Nature Research (NINA), NO-7485 Trondheim, Norway; Department of Biology, CEES & Evogene, University of Oslo, Oslo, Norway; Department of Biology, Lund University, Lund, Sweden

## Abstract

Understanding variation in rates of evolution and morphological disparity is a goal of macroevolutionary research. In a phylogenetic comparative methods framework, we present three explicit models for linking the rate of evolution of a trait to the state of another evolving trait. This allows testing hypotheses about causal influences on rates of phenotypic evolution with phylogenetic comparative data. We develop a statistical framework for fitting the models with generalized least-squares regression and use this to discuss issues and limitations in the study of rates of evolution more generally. We show that the power to detect effects on rates of evolution is low in that even strong causal effects are unlikely to explain more than a few percent of observed variance in disparity. We illustrate the models and issues by testing if rates of beak-shape evolution in birds are influenced by brain size, as may be predicted from a Baldwin effect in which presumptively more behaviorally flexible large-brained species generate more novel selection on themselves leading to higher rates of evolution. From an analysis of morphometric data for 645 species, we find evidence that both macro- and microevolution of the beak are faster in birds with larger brains, but with the caveat that there are no consistent effects of relative brain size.[Baldwin effect; beak shape; behavioral drive; bird; brain size; disparity; phylogenetic comparative method; rate of evolution.]

Rates of evolution are highly variable. While some species and traits remain nearly unchanged for tens of millions of years, others undergo dramatic shifts in mere dozens of generations (Simpson 1944; Eldredge and Gould 1972; Hendry and Kinnison 1999; Schluter 2000; Gould 2002; Hendry et al. 2008; Uyeda et al. 2011; Hunt and Rabosky 2014; Gingerich 2019; Reznick et al. 2019; Voje et al. 2020; Zelditch et al. 2020). Explaining these differences is a key challenge for a theory of macroevolution (e.g., Hansen and Houle 2004; Eldredge et al. 2005; Estes and Arnold 2007; Bell 2012; Hansen 2012; Arnold 2014; Jablonski 2017a,b). Yet, the majority of work on rates of trait evolution is descriptive and lacking in quantitative hypotheses and statistical methods derived from first principles.

While substantial progress may have to wait for a more mature quantitative theory of macroevolutionary change, it is important to move beyond mere description toward testing hypotheses about factors influencing rates of evolution. Model-based phylogenetic comparative methods provide a framework for such tests. The models that are used to translate phylogenetic relationships into the statistical covariances needed for statistical analysis usually depend on parameters related to rates of evolution. In particular, the commonly used Brownian-motion model includes trait-specific rates of “diffusion” that are estimated as a part of the analysis. These can be used to estimate rates of evolution in different traits or directions of morphospace (e.g., Felsenstein 1985; Lynch 1991; Adams 2013). Methods have been developed to estimate the Brownian diffusion rate in different parts of the phylogeny, and this allows testing for clade differences in rates of evolution (Garland 1992; Martins 1994; McPeek 1995; Gittleman et al. 1996; O’Meara et al. 2006; Thomas et al. 2006; Revell 2008; Revell and Harmon 2008; Eastman et al. 2011; Revell et al. 2011; Beaulieu et al. 2012; Adams 2014; Fuentes et al. 2016; Weir and Lawson 2016; Caetano and Harmon 2019; May and Moore 2020). Such approaches make it possible to obtain and compare estimated rates of evolution from standard comparative data (e.g., Pie and Meyer 2017; Alhajeri and Steppan 2018; Chira et al. 2018; Revell et al. 2018). Estimated rates can then be used as data in further analyses to test hypotheses about causes of rate differences, but this approach entails statistical and interpretational difficulties in dealing with estimation error and linking rates with explanatory variables. What is lacking are methods for combining rates of evolution with explanatory variables that are themselves treated as evolving entities in a common model. This would allow direct estimation of causal links in a statistical framework that accounts for stochasticity in both response and predictor variables.

Here, we explore some stochastic-process models for estimating the relationship between rates of evolution in a quantitative trait, formalized either as the diffusion parameter of a Brownian motion or as the variance of recent deviations from a phylogenetic prediction, and potential explanatory variables modeled as evolving quantitative traits on the phylogeny. We illustrate the models with an analysis of rates of evolution in bird beaks based on data from Cooney et al. (2017) and Chira et al. (2018). We relate these rates to measures of absolute and relative brain sizes, thus revisiting the classical analyses of Wyles et al. (1983) to test whether evolution is speeded up by increased behavioral flexibility due to a Baldwin effect (e.g., Baldwin 1896; Popper 1972; Wilson 1985). We argue that the classical tests of this hypothesis were inadequate, but that current data based on orders of magnitude more species, accurate phylogenies, and better statistical approaches now make it feasible to test such hypotheses.

## Theory

### Models for Testing Macroevolutionary Hypotheses about Rates

In contrast to standard models used in comparative methods for continuous traits, most of our models will not be Gaussian, meaning that the resulting distributions of the traits will not be normal (Gaussian). This complicates statistical analyses as standard statistics based on normal distributions cannot be used. Additionally, the joint probability distributions are hard to derive, and this rules out exact likelihood or Bayesian approaches. It is possible, however, to characterize the distributions by deriving moments. This allows the use of methods-of-moments estimators and generalized least squares, which do not require exact distributional assumptions. Approximate and numerical likelihood approaches are also possible but will not be explored here.

For each model, we will start by deriving moments of the trait distributions after a time interval $t$. We then use this to motivate a regression-based estimator of the influence of a predictor $x$ on the rate of evolution of a trait $y$. Because the state of a Brownian motion is uncorrelated with its own linear deviation from the starting point, this will take the form of a regression of the squared trait deviation on the predictor. We then derive the variances and covariances of the relevant functions for phylogenetically related species, and use this to characterize the residual variance matrix for the regression. This forms the basis for generalized least-squares (GLS) estimation. We present three models that are all based on the assumption that the variance of the evolutionary rate is linear in the predictor. This assumption helps generate approximately linear regressions of $y^{2}$ on $x$ and will often be a reasonable choice because independent components act additively on the variance scale. Some microevolutionary models also predict approximate additive effects of microevolutionary parameters on variances of change (Bolstad et al. 2014).

### Model 1: Predictor Evolves as a Brownian motion

In this model, the response variable, $y$, follows a Brownian motion with a rate variance that is a linear function of the predictor variable, $x$, which itself follows a standard Brownian motion with constant variance, $\sigma^{2}$. The model is

(1) \begin{align*}\label{eq1} \begin{aligned} dy &= \sqrt {a + bx} dW_1, \\ dx &= \sigma dW_2, \end{aligned} \end{align*}

where the ${\it dW}_{i}$ are uncorrelated white noise, and we assume that the initial conditions are $y_{0}=x_{0} = 0$. The influence of $x$ on the rate of evolution in $y$ is characterized by the parameter $b$, and the goal is to find an estimator of $b$. This model breaks down when $a + {\it bx}$ becomes negative, and should be seen as an approximation that may be valid for the range of $x$ values spanned by the species to be used in the analysis.

As there is no causal influence of $y$ on the evolution of $x$, the unconditional distribution of $x$ is Gaussian with mean equal to the initial condition, $x_{0}$, and variance equal to $\sigma^{2}t$, where $t$ is the time from the root to the tip of the phylogeny. The moments of $y$ and the cross-moments between $y$ and $x$ are derived in Appendix A.1 by stochastic integration. This reveals that $y$ and $x$ are indeed uncorrelated, but the squared deviation of $y$ from its mean is correlated with $x$ as

(2) \begin{align*}\label{eq2} Cov[y^2,x] = \frac{b\sigma^2t^2}{2}, \end{align*}

which suggests that a regression of $y^{2}$ on $x$ can be used to estimate the b-parameter. In Appendix B.1, we show that the best linear predictor of the species vector of $y^{2}$ from the species vector of the $x$-variable is

(3) \begin{align*}\label{eq3} \left( {a-v_y } \right){{\bf 1}} + b\left( {{{\bf I}}- \textstyle{1 \over 2}\left( {{{\bf T}} \circ {{\bf T}}} \right){{\bf T}}^{-1}} \right){{\bf x}}, \end{align*}

where ** x** is the column vector of species predictor variables, ** 1** is a column vector of ones, ** I** is the identity matrix, ** T** is the matrix of shared branch lengths between all the species pairs, $^{\circ}$ denotes Hadamard (i.e., elementwise) multiplication, and $v_{y}$ is the error variance in the prediction of the root value of the y-variable. This model assumes an ultrametric phylogeny scaled to unit height and that the $x$ and $y$ variables are centered on their predicted values. The residual variance matrix for this model is

(4) \begin{align*}\label{eq4} Var\left[ {{\bf r}} \right] &= 4av_y {{\bf T}} + 2\left( {a^2 + b^2v_x } \right){{\bf T}} \circ {{\bf T}}\nonumber\\ &+ b^2\sigma^2\left( {{{\bf T}} \circ {{\bf T}} \circ {{\bf T}}-\textstyle{1 \over 4}\left( {{{\bf T}} \circ {{\bf T}}} \right){{\bf T}}^{-1}\left( {{{\bf T}} \circ {{\bf T}}} \right)} \right), \end{align*}

where $v_{x}$ is the variance in the predicted root value used to center the x-variable. Equations 3 and 4 can be used to estimate the parameters $a$ and $b$ in an iterated GLS procedure conditionally on the phylogeny and previous estimates of the $\sigma^{2}$, $v_{y}$, and $v_{x}$. The units of the regression parameters are $[a] = [y^{2}]$ and $[b] = [y^{2}/x]$.

### Model 2: Predictor Evolves as a Geometric Brownian motion

In Model 1, we assumed that the predictor $x$ follows a Brownian motion. This yields simplicity and may be realistic in many cases, but there are also cases in which this assumption is problematic. In particular, many candidate predictor variables will be on ratio scale types. Such variables must be positive and tend to follow multiplicative dynamics, which is incompatible with Brownian motions. The usual solution to this problem is log transformation, as the logarithm of a multiplicative process may follow additive dynamics compatible with Brownian motions, but this does not solve the problem if the causal influence of the predictor variable on the rate of evolution is linear on the original scale. To deal with such situations, we use the model

(5) \begin{align*}\label{eq5} \begin{aligned} dy &= \sqrt {a + bx} dW_1, \\ dx &= \textstyle{1 \over 2}\sigma^2xdt + \sigma xdW_2, \end{aligned} \end{align*}

where $x$ follows geometric Brownian motions, which is equivalent to Brownian motions of the logarithm of $x$. We again assume that the y-variable is centered on an unbiased predictor of its root value. We also assume that the x-variable is standardized with an unbiased predictor of its logarithmic root value. In this model, $y$ is also uncorrelated with $x$ (and any power of $x)$, but after a time interval $t$,

(6) \begin{align*}\label{eq6} Cov[y^2,x] = \frac{2b}{3\sigma^2}\left( {e^{2\sigma^2t} + 2e^{\textstyle{1 \over 2}\sigma^2t}-3e^{\sigma^2t}} \right), \end{align*}

which shows that the b-parameter can be estimated from a regression of the squared y-variables on the x-variables. In Appendix B.1, we show that the best linear predictor of the vector of squared y-variables from the vector of x-variables is

(7) \begin{align*}\label{eq7} \left( {A-v_y } \right)1 + \frac{2b}{\sigma^2}\left( {{{\bf I}} - \frac{2}{3}e^{-\frac{1}{2}\sigma^2}\left[{e^{\frac{3}{2}\sigma^2t_a }-1} \right]\left[ {e^{\sigma^2t_a }-1} \right]^{-1}} \right){{\bf x}}, \end{align*}

where ** x** is centered on its predicted mean (see Appendix B.1), [f($t_{a}$)] denotes a matrix in which the ij’th element is f($t_{a}$) with $t_{a}$ being the shared branch length between species $i$ and $j$ on the phylogeny, and $A$ is an intercept equal to $a$ minus a correction term given in the appendix. We have again assumed an ultrametric phylogeny scaled to unit height ($t = 1$).

The residual variance matrix for this model is

(8) \begin{align*}\label{eq8} \begin{aligned} Var\left[ {{\bf r}} \right] &= 4av_y {{\bf T}} + \frac{8bv_y e^{\textstyle{1 \over 2}v_{\ln x} }}{\sigma^2}\left[ {e^{\textstyle{1 \over 2}\sigma^2t_a }-1} \right]\\ &\quad + 2a^2{{\bf T}} \circ {{\bf T}} + \frac{8abe^{\textstyle{1 \over 2}v_{\ln x} }}{\sigma^2}{{\bf T}} \circ \left[ {e^{\textstyle{1 \over 2}\sigma^2t_a }-1} \right] \\ &\quad + \frac{2b^2e^{2v_{\ln x} }}{\sigma^4}\left( \frac{8}{3}\left[ {e^{2\sigma^2t_a }-e^{\textstyle{1 \over 2}\sigma^2t_a }} \right]- \left[ {e^{2\sigma^2t_a }-1} \right]\right.\\ &\quad \left.-\frac{8}{9}\left[ {e^{\textstyle{3 \over 2}\sigma^2t_a }-1} \right]\left[ {e^{\sigma^2t_a }-1} \right]^{ - 1}\left[ {e^{\textstyle{3 \over 2}\sigma^2t_a }-1} \right] \right), \end{aligned} \end{align*}

where $v_{lnx}$ is the error variance in predicting ln$x_{0}$, the root value of the x-variable on log scale. Note that $\sigma^{2}$ for this model is the predicted variance of the natural logarithm of $x$ across the tips of an ultrametric phylogeny scaled to unit height.

The regression is not linear in the sense that the covariance of $y^{2}$ with higher powers of the predictor are not zero, but these tend to zero as $\sigma^{2}$ increases. In the simulations, however, we found some bias when $\sigma^{2} = 2$, but not for $\sigma^{2} \le $ 1. Nevertheless, if $\sigma^{2}$ is below 0.5 or so, it may be better to use the simpler Model 1 since the multiplicative dynamics of a ratio-scale variable with moderate variation can be approximated with additive dynamics and Brownian motions.

### Model 3: Predictor Affects Rates of Recent Microevolution

In a large data set of morphological traits, Uyeda et al. (2011) observed that recent microevolution was largely decoupled from macroevolution in a so-called “blunderbuss” pattern. The best general model for this pattern was a combination of a white noise with a million-year-scale Gaussian point process. Gaussian point processes converge to Brownian motions on long time scales, and the current phenotype of a species may thus be reasonably described by the sum of a Brownian motion on a macroevolutionary scale and a white noise representing recent evolution on a less than million-year time scale. In contrast to the previous models, which represent rates of cumulative macroevolution over the entire phylogeny, the following model may be used to test if rates of recent microevolution are related to candidate predictor variables. In light of the blunderbuss, we can do this by using the squared deviation of each species from its macroevolutionary prediction as a response variable.

We model the species trait vector as ${\bf y} ={\bf y}_{\rm macro} + {\bf y}_{\rm micro}$, where ${\bf y}_{\rm macro}$ is the result of a macroevolutionary process (e.g., Brownian motion) unfolding on the phylogeny and ${\bf y}_{\rm micro}$ is a microevolutionary deviation, independent of the macroevolutionary process, but with a variance that may depend on the predictor variable as

(9) \begin{align*}\label{eq9} {{\bf y}}_{\rm micro} \sim N\left( {{{\bf 0}},a{{\bf I}} + diag\left( {b{{\bf x}}} \right)} \right), \end{align*}

where **x** is a vector of species-specific predictor variables. The *diag*-function applied to a vector yields a diagonal matrix with the vector along the diagonal. In this case, only the recent value of the predictor matters and we will assume that it does not interact with the macroevolutionary process.

As the microevolutionary deviation is not directly observable, we model it as ${{\bf y}} - \hat{\bf y}$, where ${\hat{\bf {y}}}$ is a vector of macroevolutionary predictions for each species based on the other species given the phylogeny and a model of macroevolution. The best linear unbiased predictor for each species based on the other species in the phylogeny is the vector

(10) \begin{align*}\label{eq10} {\hat{\bf y}} = {\bar{\bf y}} + \left( {{{\bf V}} -d({{\bf V}}^{-1})^{-1} } \right){{\bf V}}^{-1}\left( {{{\bf y}} - \bar{\bf y}} \right), \end{align*}

where the $d$-function sets to zero all nondiagonal elements of its argument, **V** is the variance matrix of the ** y** vector, and ${{\bf \bar {y}}}$ is the mean vector. Technically, the elements of the mean vector should be calculated separately for each species by not including that species, but in practice with many species it makes little difference to use the same grand mean for all species. Any fixed effects of the evolutionary model can be included in the mean vector. This equation can be used to make predictions from any evolutionary model by using the phylogenetic variance matrix and mean vector derived from that model. It can be derived from the general equation for best linear unbiased prediction of ancestral states given in Martins and Hansen (1997) to predict the trait value of an hypothetical species in the exact phylogenetic position of the (deleted) species to be predicted. The form of the equation can be verified by elementwise comparison. The deviance from the prediction is then

(11) \begin{align*}\label{eq11} {{\bf y}} - {\hat{\bf y}} = d({{\bf V}}^{-1})^{-1}{{\bf V}}^{-1}\left( {{{\bf y}} - {\bar{\bf y}} } \right), \end{align*}

and the squares of the elements of this vector can be used as a response variable in the regression model. If the evolutionary process is Gaussian, then these deviations are normally distributed with mean zero and variance matrix

(12) \begin{align*}\label{eq12} Var\left[ {{{\bf y}} - {\hat{\bf y}} } \right] = \left( {d({{\bf V}}^{-1}){{\bf V}}d({{\bf V}}^{-1})} \right)^{ -1}. \end{align*}

The variance matrix of the y-vector is

(13) \begin{align*}\label{eq13} {\bf V} = {\bf V}_{\rm macro} + {\bf V}_{\rm micro} + {\bf V}_{\rm mesurement}, \end{align*}

where ** V**$_{\rm macro}$ is the phylogenetic variance matrix derived from the assumed macroevolutionary process, ** V**$_{\rm micro} (= a{\bf I}+{\it diag(b}{\bf x}))$ is the variance matrix contributed by recent microevolution (white noise) and ** V**$_{\rm measurement}$ contains any measurement variances we wish to add to the model. If we assume that macroevolution is a Brownian-motion process, ** V**$_{\rm macro}= \sigma_{y}^{2}$** T**, where ** T** is the matrix of shared branch lengths, but other Gaussian processes, such as an Ornstein–Uhlenbeck process, may also be used.

In this model, the microevolutionary parameters $a, b$, and macroevolutionary parameters such as $\sigma_{y}^{2}$ could be estimated with maximum likelihood based on the normal distribution, but in line with the approach of the other models, we focus on a GLS regression of the square of the predicted microevolutionary deviations on the predictor variable as

(14) \begin{align*}\label{eq14} {\bf A} + b\left( {\left( { d\left( {{{\bf V}}^{-1}} \right)^{-1}{{\bf V}}^{-1}} \right) \circ \left( { d\left( {{{\bf V}}^{- 1}} \right)^{-1}{{\bf V}}^{-1}} \right)} \right)\left( {{{\bf x}}-{\bar{\bf x}}} \right) \end{align*}

as shown in Appendix B.1. The intercept vector is

(15) \begin{align*}\label{eq15} {\bf A} &= \textit{diag}\left[ {d\left( {{{\bf V}}^{-1}} \right)^{- 1}{{\bf V}}^{-2}d\left( {{{\bf V}}^{-1}} \right)^{-1}} \right]a\nonumber\\ &\quad + \textit{diag}\left[ {d\left( {{{\bf V}}^{-1}} \right)^{-1}{{\bf V}}^{- 1}{{\bf V}}_{\rm macro} {{\bf V}}^{-1}d\left( {{{\bf V}}^{-1}} \right)^{-1}} \right] \end{align*}

which is computed by use of the relation ${{\bf y}} - {\hat{\bf {y}}} = {\bf y}_{\rm micro} + ({\bf y}_{\rm macro}- {\hat{\bf {y}}})$, and measurement variance can be added to **V**$_{macro }$ This assumes that micro- and macroevolution are independent, and that the predictor variable only affects the microevolutionary deviation. The latter assumption is not likely to be important in the GLS setting, as the effects of macroevolution are largely removed by using the deviation from the macroevolutionary prediction as a response variable. The units of these parameters are $[a] = [y^{2}]$ and $[b] = [y^{2}/x]$.

Because predictions for the different species are correlated with each other, we need to compute the residual covariances. Assuming that ${{\bf y}} - {\hat{\bf{y}}}$ is normally distributed with zero mean, the vector of their squared elements follows a multivariate $\chi^{2}$-distribution with one degree of freedom and covariances

(16) \begin{align*}\label{eq16} &Cov\left[\left( {y_i-\hat{y}_i}\right)^2\left( {y_j-\hat{y}_j}\right)^2 \right] =2 Cov\left[y_i-\hat{y}_i, y_j-\hat{y}_j\right]^2\nonumber\\ &\quad =2[d({\bf V}^{-1}){\bf V}d({\bf V}^{-1})]^{2}_{ij} \end{align*}

Because the predictor variable, $x$, is regarded as fixed, these covariances are also the residual covariances of the regression model. In matrix notation the residual variance matrix can then be written

(17) \begin{align*}\label{eq17} Var[{{\bf r}}] = 2\left( {d({{\bf V}}^{-1}){{\bf V}}d({{\bf V}}^{-1})} \right)^{-1} \circ \left( {d({{\bf V}}^{-1}){{\bf V}}d({{\bf V}}^{-1})} \right)^{-1}. \end{align*}

## Methods

### The Statistical Model

To analyze these models we will use the standard framework of phylogenetic generalized least squares (e.g., Martins and Hansen 1997). The statistical model is

(18) \begin{align*}\label{eq18} {\bf Y} = {\bf D}\boldsymbol\beta +{\bf r}, \text{Var}[{\bf r}] = {\bf V}_{\bf r}, \end{align*}

where ** Y** is a vector of species observations, which in our case will be the species squared deviation from a grand mean (Models 1 and 2) or prediction (Model 3). The ** D** is a design matrix with predictor variables, $\boldsymbol{\beta}$ is a vector of parameters to be estimated, and ** r** a vector of residuals with mean zero and, a not necessarily diagonal, variance matrix, ** V**$_{\bf r}$. If the ** D** and ** V**$_{\bf r}$ are specified, generalized least squares (GLS) estimates can be used to obtain best linear unbiased estimates of the parameter vector $\boldsymbol{\beta}$, as

(19) \begin{align*}\label{eq19} \boldsymbol{\hat{\beta}} = \left( {{{\bf D}}^T{{\bf V}}_r^{-1} {{\bf D}}} \right)^{-1}{{\bf D}}^T{{\bf V}}_r^{-1} {{\bf Y}}, \end{align*}

with estimation variance

(20) \begin{align*}\label{eq20} Var\left[ \boldsymbol{\hat{\beta}} \right] = \left( {{{\bf D}}^T{{\bf V}}_r^{-1} {{\bf D}}} \right)^{-1}, \end{align*}

which can be used to form standard errors. Note that these standard errors cannot be translated into confidence intervals when the distribution of the residuals is unknown. In our models, the ** V**$_{\bf r}$ matrix will be a function of model parameters such as $a, b$ and $\sigma^{2}$. The parameter $\sigma^{2}$ in Models 1 and 2 can be obtained by maximum likelihood based on the predictor variable alone (since either the predictor or its logarithm is assumed to be normally distributed). The other parameters can be estimated with an iterated procedure starting from an initial guess (e.g., based on ordinary least-squares regression). Usually, such schemes converge in a handful of iterations.

The design matrix, ** D**, will normally contain two columns. For Model 1, we use

(21) \begin{align*}\label{eq21} {{\bf D}} = \left[ {{{\bf 1}}, \left( {{{\bf I}}-\textstyle{1 \over 2}\left( {{{\bf T}} \circ {{\bf T}}} \right){{\bf T}}^{-1}} \right){{\bf x}}} \right], \end{align*}

with ** x** centered on its predicted root value. This estimates $\boldsymbol{\hat{\beta}}=\{\hat{A},\hat{b}\}$, and an unbiased estimator of $a$ can be obtained by adding $v_{y}$ to $A$. For Model 2, we use

(22) \begin{align*}\label{eq22} {{\bf D}} = \left[ {{{\bf 1}}, \frac{2}{\sigma^2}\left( {{{\bf I}}-\textstyle{2 \over 3}e^{-\textstyle{1 \over 2}\sigma ^2}\left[ {e^{\textstyle{3 \over 2}\sigma^2t_a }-1} \right]\left[ {e^{\sigma^2t_a }-1} \right]^{-1}} \right){{\bf x}}} \right], \end{align*}

with ** x** standardized with its predicted root value and centered on its predicted mean value. Here, we estimate $a$ by adding $v_{y}$ plus a bias-correction term given in appendix B to the intercept. For Model 3, we use

(23) \begin{align*}\label{eq23} {\bf D} &=[\text{diag(d}({\bf V}^{-1})^{-1}{\bf V}^{-2}\text{d}({\bf V}^{-1})^{-1}),\nonumber\\ &\quad(\text{d}({\bf V}^{-1})^{-1}{\bf V}^{-1}) \circ (\text{d}({\bf V}^{-1})^{-1}{\bf V}^{-1}){\bf x]}, \end{align*}

with ** V** as in (13), and with the term diag(d(** V**$^{-1})^{- 1}{\bf V}^{-1}{\bf V}_{\rm macro}{\bf V}^{-1}$d(** V**$^{-1})^{-1})$ subtracted from the response vector (i.e., $a$ is estimated according to equation 15). It is also possible to add columns to these design matrices to account for various fixed effects that may influence the squared deviations.

Most of our models require that variables are centered on or standardized with predicted values at the root of the phylogeny. Under the assumption of Brownian motion, the expression

(24) \begin{align*}\label{eq24} m = \frac{{{\bf 1}}^T{{\bf T}}^{-1}{{\bf y}}}{{{\bf 1}}^T{{\bf T}}^{-1}{{\bf 1}}}, \end{align*}

will be an unbiased predictor of the root value of $y$. Its variance is $v_{y}=a/n_{e}$, where $a$ is the variance parameter of the Brownian motion, and $n_{e}={{\bf 1}}^T{{\bf T}}^{-1}{{\bf 1}}$ is an effective sample size. The same equation can be used for the root value of $x$ or ln$x$ as needed. In principle better predictors could be found by replacing ** T** with the estimated variance matrices of the variables, but this is unlikely to make a difference in practice.

The residual variance matrix, ** V**$_{\bf r}$, will contain a biological component with residual variances and covariances as described in equation 4 for Model 1, equation 8 for Model 2, and equation 17 for Model 3. When available, measurement or observation variances for response and predictor variables may also be included in the residual variance matrix and used to correct for attenuation as described in Appendix B.1. Observation variances in comparative studies are often available from standard errors or confidence intervals of the means (or other statistics) used from the individual species. Note that the observation variance of the response variable in this case would be the predicted variance of the square of the mean. If we assume that the observation error is normally distributed, observation variances of squared means can be obtained as 2*se*$^{4}$, where *se* is the standard error of the mean. Hansen and Bartoszek (2012), Garamszegi (2014), and Grabowski et al. (2016) provide further discussion of observation error in comparative studies.

The three models are set up to explain the variance of a variance, and we expect a worse fit as compared to standard linear models of trait means. Indeed, even an $R^{2}$ of a few percent could be regarded as a strong signal in these models. To illustrate, let us assume that, as in all our models, the $y$ variable conditional on $x$ is normally distributed with a variance that is linear in $x$, as in Var$[y|x] = a + {\it bx}$. Then, assuming that $y$ is mean centered, the proportion of variance in the square of $y$ explained by $x$ is

$$
\begin{align*}
R^2 &= \frac{Var[E[y^2|x]]}{Var[y^2]} = \frac{Var[E[y^2|
x]]}{E[Var[y^2|x]] + Var[E[y^2|x]]}\\
&= \frac{b^2Var[x]}{2a^2 +
4ab\bar {x} + 2b^2\bar {x}^2 + 3b^2Var[x]} < \frac{1}{3}.
\end{align*}$$

Hence, with these assumptions there is a theoretical ceiling for $R^{2}$ at less than 1/3. In practice, much less variance will be explained. For example, in a rather ideal situation with two equal-sized groups of species with one group having twice the variance of the other, we get an expected $R^{2} = 1/21 \approx 5{\%}$ for the simple ANOVA set up (i.e., $a = 1, b = 1$, and Var$[x] = 1/4$ with $x$ as a zero-one indicator variable); even with infinitely larger variance in one group ($b=\infty )$ the variance explained is limited to $R^{2} = 1/5$.

### Step-by-Step Summary of Approach.

1. Do exploratory analysis of both the response ($y$) and predictor ($x$) variable to assess their distributional properties and approximate modes of evolution. Phylogenetic signal can be assessed by estimating a phylogenetic heritability (Lynch 1991; Housworth et al. 2004) or a phylogenetic halflife (Hansen 1997). This will tell if the variables (or their logarithms) can be approximated by a Brownian motion and help decide which of the three models is most appropriate.

2. Prepare the data by choosing appropriate scales and units for the analysis to be used. This may involve log transformation and centering on the mean or predicted root value. Be careful about variance standardization as this may obscure the signal of interest.

3. Formulate the hypothesis to test, and pay attention to the form of the relationship between predictor and rates that is predicted by the hypothesis.

4. Fit the predictor variable or its logarithm to a Brownian motion to estimate the parameter $\sigma^{2}$. In cases of low but nonzero phylogenetic signal, it is possible to fit a mixed model and use the rate parameter ($\sigma^{2}$) from the Brownian-motion component.

5. Choose the model to use. This should be based both on statistical considerations (distribution of variables, model adequacy, e.g., Pennell et al. 2015) and biological considerations (likely or hypothesized modes of evolution and relations between variables). Model 2 should only be used with predictor variables on ratio or log-ratio scale types (see Houle et al. 2011), and log-transformed predictors would not be suitable for this model. Note that Model 3 is testing a different hypothesis than Models 1 and 2. In cases of low phylogenetic signal, Model 3 would be fine to use and will then approximate an ordinary least-squares regression, but Models 1 and 2 would be problematic to interpret as they assume the relationship between the variables is built gradually over the history of the species.

6. Fit the desired models as described above.

7. Calculate the parameters $a$ and $b$ from the regression parameters, and report results with units and estimated uncertainty. Interpret effect sizes. The $R^{2}$ of the model should be considered, but be aware that we can only reasonably expect to explain a few percent of the variance. Resampling procedures can be used to further explore model adequacy and uncertainty of the estimates.

We have implemented statistical machinery for most of the operations in Step 4–7 in the function rate_gls in the R-package evolvability (Bolstad et al. 2014). We have also included functions for simulation of the three evolutionary models (function: simulate_rate), parametric bootstrapping (function: rate_gls_boot), and a vignette explaining the use of the functions (Analyzing rates of evolution). The package is available at CRAN with the latest version at “github.com/GHBolstad/evolvability.”

## Application

### Do Beak Shapes Evolve Faster in Large-Brained Birds?

Wyles et al. (1983) argued that animals with larger brains have faster rates of morphological evolution than comparable animals with smaller brains. They attributed this to behavioral drive, a process by which learning allows individuals to explore new ways of life and thereby setting up novel selection pressures on their anatomy and physiology that lead to more extensive divergence on macroevolutionary time scales. This idea is part of a broader conglomerate of hypotheses and theories about how plasticity, learning, preferences, intentions, culture, and niche construction can influence evolution and adaptation by altering selection pressures or allowing organisms to persist in novel environments long enough to adapt to them (e.g., Baldwin 1896; Popper 1972; Wake et al. 1983; Odling-Smee et al. 2003; Frank 2011; Zuk et al. 2014). The effects of such mechanisms on rates of evolution are far from obvious, however, and it has also been argued that behavioral flexibility may buffer against changes and help explain morphological stasis (e.g., Bogert 1949; Wake et al. 1983; Hansen and Houle 2004). Paenke et al. (2007) show that both scenarios are theoretically plausible and provide general conditions for plasticity to accelerate or decelerate the response to selection.

The study of Wyles et al. (1983) was based on comparing morphological disparity between a few large clades with different degrees of encephalization such as primates against other mammals, song birds against other birds, and mammals against reptiles. This procedure yields only a handful of comparisons and is vulnerable to spurious third-variable effects and artifacts of measurement stemming from the many biological differences that must exist at such broad phylogenetic scales. It can only be regarded as crudely suggestive.

### Application: Methods

To illustrate our models, we capitalize on recent large-scale phylogenies and open-source data bases for bird morphology to test the hypotheses of Wyles et al. (1983) and Wilson (1985) more rigorously. We use 3D morphometric beak-shape data taken from Cooney et al. (2017) and brain- and body-size data from Fristoe et al. (2017) and Tsuboi et al. (2018) for 651 bird species to test if rates of beak-shape evolution are influenced by brain size. We excluded the paleognaths, comprising four species of ratites and one species of tinamou, from the analysis, as this group is both a sister group to the rest and biologically different from most other birds. We also removed the emperor penguin (*Aptenodytes forsteri*), as this species was a strong outlier in terms of both brain and body size. This left us with 645 species that we used for the analyses presented here. Results were qualitatively similar when including all species.

The 3D-landmark data (4 landmarks and 75 semilandmarks) of beak shape were standardized by generalized Procrustes analysis before principal-component analysis in Cooney et al. (2017). We studied the first eight principal components from this analysis, which collectively explain more than 99% of the variance. The principal components are in units of centroid size (i.e., mean linear distance of landmarks to centroid). The phylogeny was taken from Jetz et al. (2014), and we used a maximum credibility tree from a sample of thousand phylogenies based on family-level topologies from Ericson et al. (2006) using TreeAnnotator version 2.4.5.

**Figure 1.**
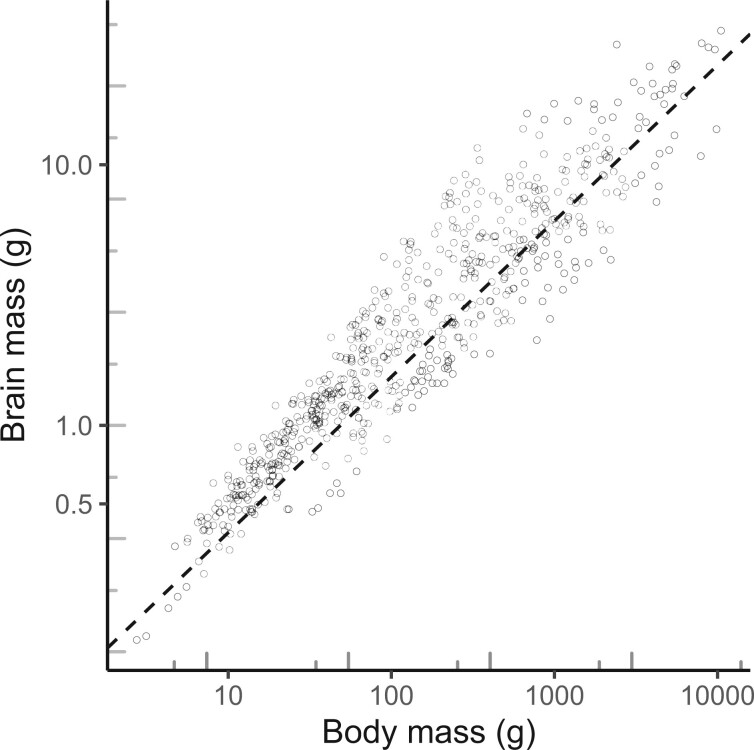
Brain–body allometry for the 645 species of birds used in this study. The data points are species means taken from Tsuboi et al. (2018). The regression line $-2.21 (\pm 0.12) + 0.598 (\pm 0.008)$ log(body mass), $R^{2} = 88.4{\%}$) was estimated from a “direct-effect” Ornstein–Uhlenbeck model and corrected for estimation error in species means (including attenuation) as described in Grabowski et al. (2016). The phylogenetic half-life and stationary variance of the Ornstein–Uhlenbeck model were $t_{1/2} = 5$$h$ with 2-unit support interval: (1.35–$\infty$) and $\sigma^{2}_{ou} = 0.43$ with 2-unit support interval: (0.14–0.49). The tree length was $h = 104$ myr. The long half-life means that the residuals evolve similarly to a Brownian motion.

We tested for the effects of both absolute and relative brain mass. To obtain relative brain mass we performed an allometric analysis of brain mass on body mass (Fig. 1) with the R-package slouch (Hansen et al. 2008; version 2.0.0. documented in Kopperud et al. 2020). Following Grabowski et al. (2016), we fitted a direct-effect model and accounted for estimation error in species means based on reported standard errors of the means of logged brain and body masses. The estimated allometric exponent corrected for attenuation due to observation error was $3/5 (= 0.60 \pm 0.01)$, and log body size explained 88% of the variance in log brain size. Relative brain mass is based on the residuals from this allometric model.

The basic statistics of the traits are in Table 1. The brain- and body-size data show a strong phylogenetic signal and good fit to Brownian motion on log scale. For the beak-shape data, the first principal component, which is an axis of stoutness to pointedness of the beak, likewise show strong phylogenetic signal and good fit to Brownian motion. The other seven “shape” traits show a moderately strong phylogenetic signal, leaving room for Ornstein–Uhlenbeck-type dynamics or a component of recent evolution. The two-unit support intervals for the phylogenetic halflife exclude both infinity (Brownian motion) and zero (white noise) for all these.

**Table 1.** Basic statistics for the among-species data: the phylogenetic heritability, ${\it PH}^{2}$, is the fraction of among-species variance attributable to Brownian motion (Lynch 1991). The $\sigma^{2}_{\rm Brownian}$ is the rate variance of the Brownian motion in units per tree length ($h = 104$ my), and $\sigma^{2}_{\rm residual}$ is the nonphylogenetic residual variance. The phylogenetic halflife, $t_{1/2}$, is the time in units of tree length to lose half the expected ancestral influence in an Ornstein–Uhlenbeck model of evolution (Hansen 1997). Body and brain size are in units of ln (gram), and principal components of beak shape in units of 100 $\times $ centroid size (cs). The variances are in units of the squares of the trait units. The phylogentic heritability and the variance components were estimated with the function Almer in the evolvability package, and the halflife was estimated with slouch.

Trait	$PH^{2}$	$t_{1/2}$	$\sigma^{2}_{\rm Brownian}$	$\sigma^{2}_{\rm residual}$
Log body mass	0.97	4.5$^{a}$	2.54	0.082
Log brain mass	0.99	$\sim \infty^{a}$	1.12	0.012
Relative brain size	0.91	2.2$^{a}$	0.091	0.0095
Beak shape pc1	0.95	8.2	289	15
Beak shape pc2	0.76	0.37	156	48
Beak shape pc3	0.88	0.57	43	6.0
Beak shape pc4	0.88	0.75	20	2.9
Beak shape pc5	0.88	0.55	12	1.7
Beak shape pc6	0.75	0.34	5.0	1.7
Beak shape pc7	0.83	0.54	2.7	0.56
Beak shape pc8	0.69	0.27	1.6	0.75

$^{a}$ Corrected for estimation error in species means.

We tested the hypotheses that either absolute or relative brain size influence the rate of evolution of beak shape, and whether this happens on average throughout the phylogeny (Models 1 and 2) or in terms of recent “within-species” microevolution (Model 3). The models were fitted with the functions in the evolvability package as described above. Estimates are shown $\pm $*se* if not otherwise indicated. Reported standard errors are based on equation 20. With some exceptions, these were similar to bootstrap estimates (see Supplementary material). The predictor variables in the plots are phylogenetically weighted values that account for correlation with related species (i.e., values correspond to the entries in the design matrices in equations 21–23).

### Application: Results

**Figure 2.**
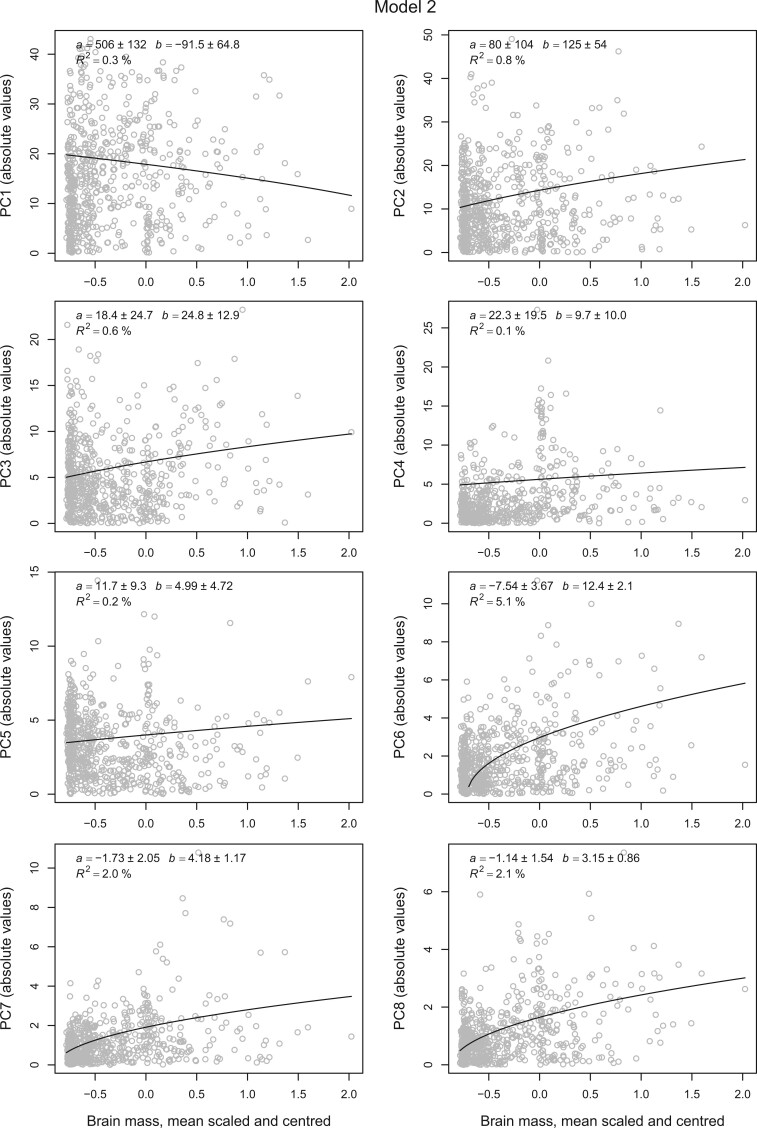
The effect of absolute brain size on beak-shape evolution. The plot shows the magnitude of the deviance from clade mean of beak-shape variables in units of centroid size ($\times 100$) plotted against (phylogentically weighted) log brain mass for 645 bird species. The fitted line and parameter estimates ($\pm $ SE) are based on Model 2 in the main text assuming that brain mass evolves like a geometric Brownian motion. The estimated slopes ($b)$ of PCs 2, 6, 7, and 8 are statistically significant in the sense that they are more than two standard errors above zero.

Starting with absolute brain mass, we note that this is a ratio-scale variable that we expect to evolve on a multiplicative scale. This makes the geometric Brownian-motion Model 2 is the most natural choice, and the mean-scaled variance for brain mass of $1.19 (= \sigma^{2}t) > 1$ means that Model 1 may be less good on an untransformed scale. The results from this model mix small to moderate positive effects of absolute brain size on the rates of evolution in most shape variables (PCs 2, 3, 7, 8) with a large positive effect on PC 6, and essentially no effects on PCs 1, 4, and 5 (Fig. 2). The phylogenetic $R^{2}$ of the positive effects are small in absolute terms, 5% for PC 6 and 0.6–2% for the others, but still give room for substantial influence on rates. The predicted rate variance, in units of squared centroid size (*cs*) per tree length ($h = 104$*my*), for PC 6 is $-7.5 \times 10^{-4} + 12.4\times 10^{-4} x$, where $x$ is mean-standardized brain mass, which means that a doubling of brain mass from the phylogenetic mean ($x =1$) would increase the rate variance from $4.9 \times 10^{-4} {\it cs}^{2}/h$ to $17.3 \times 10^{-4} {\it cs}^{2}/h$. This means that expected variance (disparity) in this shape variable of a clade with twice the average brain mass would be expected to be 3.5 times as large as the average. In other words, doubling the brain mass of a lineage would increase the expected change in PC 6 over an arbitrary time period, and thus the classic rate of evolution (i.e., the *darwin*), with a factor of $\sqrt {3.5} $, or 88%. Similar calculations for PCs 2, 3, 7, and 8 yield factors of, respectively, 27%, 25%, 65%, and 60%.

An alternative is to use Model 1 with log brain mass as predictor variable. This is equivalent in terms of its assumptions about the evolution of brain mass, but differs in that the assumed causal link to the rate variance is now linear on a proportional scale (i.e., linear on log brain mass rather than linear on brain mass). The results of this analysis are congruent with the fitting of Model 2 in that the strongest effect is for PC 6, but both this and the other effects are weaker (Supplementary Fig. S1). For PC 6 the predicted rate variance is $9.5 + 10.5$ log brain mass, which means that doubling brain mass from the clade mean ($x = 0$) by setting log brain mass to $x =$ ln 2 would increase the rate variance from $9.5 \times 10^{-4}{\it cs}^{2}/h$ to $17 \times 10^{-4} {\it cs}^{2}/h$, or the expected rate of evolution with a factor of 34%. The other “significant” effects, on PC 7 and 8, correspond to increases of 32% and 25%, respectively.

**Figure 3.**
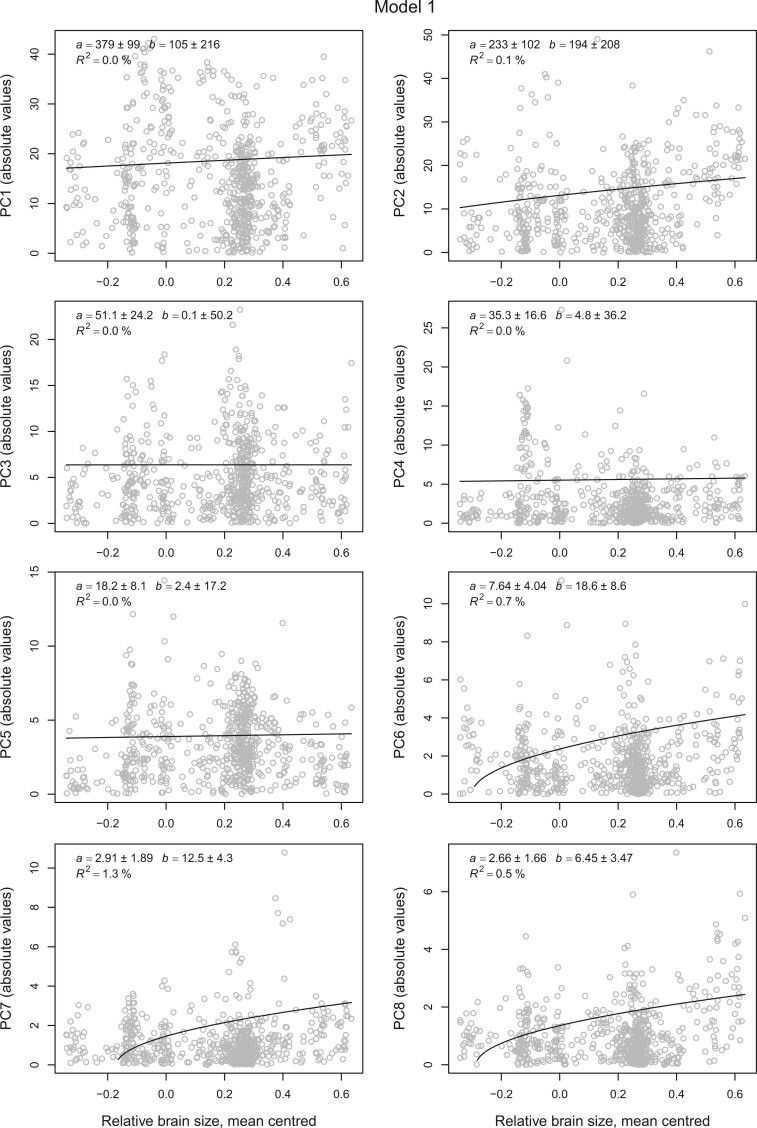
The effect of relative brain size on beak-shape evolution. The plot shows the magnitude of the deviance from clade mean of beak-shape variables in units of centroid size ($\times $100) plotted against relative brain size for 645 bird species. The fitted line and parameter estimates are based on Model 1 in the main text assuming that relative brain size evolves like a Brownian motion. The estimated slopes ($b$) of PCs 6 and 7 are statistically significant in the sense that they are more than two standard errors above zero.

For relative brain mass, which is on an interval scale type, the Brownian-motions-based Model 1 is the natural choice. Fitting this model to the beak-shape variables shows little effect (Fig. 3). The estimated slopes are all positive, but small, with phylogenetic $R^{2}$ below 1% in all cases except for PC 7. Taken in isolation the results on PC 6, 7, and 8 are consistent with some effect, but we interpret the results collectively as evidence against any substantial effect of relative brain size on rates of beak-shape evolution.

**Figure 4.**
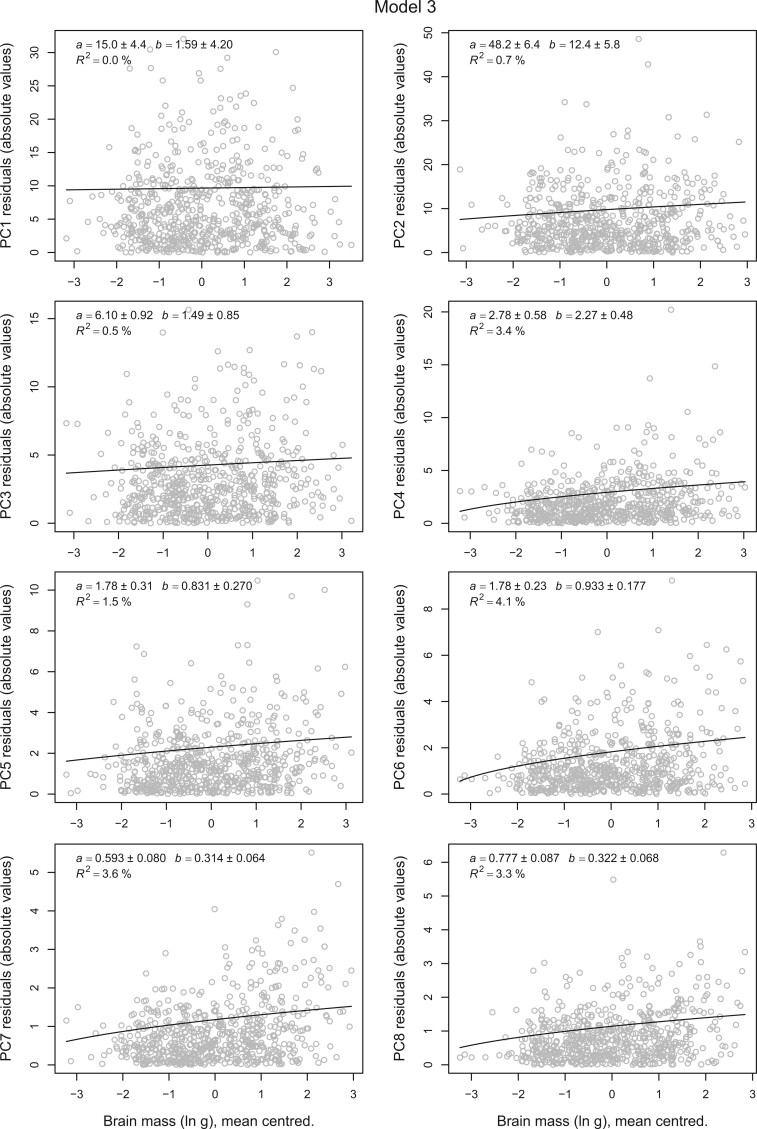
The effect of log brain size on recent beak-shape evolution. The plot shows the magnitude of the deviance of beak-shape variables from the macroevolutionary prediction in units of centroid size ($\times $100) in Model 3 plotted against (predicted) log brain mass for 645 bird species. The fitted line and parameter estimates are based on Model 3 in the main text, and the intercept used for plotting is the average of the prediction in equation 15. The estimated slopes ($b$) of PCs 2 and 4 to 8 are statistically significant in the sense that they are more than two standard errors above zero.

Fitting Model 3 tests a different hypothesis, namely that the rate of recent (within-species) evolution of beak shape is influenced by brain size. We used a Brownian-motion-based mixed model to predict the states of the shape variables at the tips of the phylogeny and used the squared deviance of the observed states from these predicted states as response variables in the regression. This model makes no assumptions about the evolution or distribution of the predictor variables, which can then be transformed and fitted on any scale that is deemed biologically reasonable. As with Model 1 there are only tiny, inconsistent effects of relative brain size (Supplementary Fig. S2), but increasing absolute brain size elevates the rates of all PCs except the first and marginally the third with $R^{2}$ ranging from 0.5% to 4% (Fig. 4). The lack of an effect on PC 1 is perhaps not surprising given that the strong phylogenetic signal of this variable leaves a lesser fraction of “recent” variation to be explained. The estimates from the other shapes indicate that the predicted residual variance at the tip would increase from 18% to 57%, corresponding to 9% to 25% increases in conventional rates of evolution.

The general lack of effect of relative brain size may suggest that the effects of absolute brain size may be caused by body size. For this reason, we also tested body mass as an explanatory variable. With Models 1 and 2, we found generally weaker effects of body mass than we found with brain mass although 3.4% of the variance in PC 6 was explained with the geometric Brownian-motion Model 2 (results not shown). This is still weaker than the effect of brain mass, but it is possible that body size may explain some of the large effect of brain mass on PC 6 in particular. For the recent-evolution model, however, the effects of body mass were similar to those of brain mass (Supplementary Fig. S3).

## Discussion

The variation in tempo and mode of evolution is such that even the basic concept of a rate of evolution defined as character change per time, as in the classic *darwin* and *haldane* measures, is problematic. Beyond a few generations, evolutionary change rarely resembles a deterministic trend, but instead becomes stochastic with huge variations in both rate and direction (e.g., Gingerich 1983, 2019; Bookstein 1987; Hunt 2012; Voje 2016). These variations are such that one cannot compare rates of change across different time intervals; in fact, below species-level time scales the amount of change is often independent of time span (Gingerich 1983, 1993, 2001, 2009; Roopnarine 2003; Uyeda et al. 2011; Voje et al. 2018) rendering conventional rates meaningless. A relationship between change and time span appears at longer, among-species, time scales, but change remains stochastic and often with scaling-relationships resembling Brownian motion (Uyeda et al. 2011). This means that rates of macroevolution cannot be measured on the scale of the trait. Instead, it is necessary to measure rates as “intensities,” variance of change per time. Over the last decades, it has become common to use the diffusion parameter of a fitted Brownian motion as a measure of rate of evolution on macroevolutionary time scales (e.g., Hunt and Carrano 2010, and references in the introduction).

Estimating rates as variances of change has a downside as compared to the estimation of a trend in that it is less statistically efficient and demands larger samples to reach comparable levels of accuracy (e.g., Slater and Pennell 2014). Our theoretical analysis illustrates this point. Variables affecting rates of evolution are not expected to correlate with the state of the evolving traits in the absence of directional trends. Correlations only appear with disparity on the scale of the squared traits and statistical analysis of causal effects on the tempo of evolution must be conducted on this scale. We have shown that even strong effects on the rates of change only generate weak correlations with the causal variable implying that we do not expect to explain more than a few percent of the among-species variance in squared deviations. This means that large samples of species are necessary to detect an effect. Fortunately, databases of morphological traits and phylogenetic relationships are accumulating and it is becoming increasingly possible to do comparative analyses with the hundreds of species necessary to test hypotheses about causal effects on rates of evolution.

Despite the mathematical complexity, the underlying idea of our method is simple: The effect of a variable on rates of evolution can be detected by a regression of squared trait deviance on the variable. The mathematics yields two refinements to a naive regression of this type. First, it shows how we can translate the coefficients of the raw regression to effects on underlying rates of evolution (the $a$ and $b$ parameters), and second, it shows how to calculate residual covariances so as to use statistically more efficient GLS estimation that accounts for phylogenetic relatedness. As with most comparative methods, both interpretation and the optimal statistical model depend on assumptions about the underlying evolutionary process. Our three models differ in these assumptions and apply under different circumstances.

### Do Large-Brained Birds Evolve Faster?

We have illustrated our methods with a data set of 645 species of birds from Cooney et al. (2017), which we used to test the hypothesis that brain size influences rates of evolution in beak shape. This hypothesis, derived from Wyles et al. (1983), may be motivated as the outcome of a Baldwin effect in which presumptively more behaviorally flexible larger-brained species are more able to discover new sources of food or novel uses of their beaks, which may introduce more frequent novel patterns of selection acting on their beaks and thus more frequent evolutionary changes in morphology. This is consistent with the recent finding of increased speciation rates in large-brained birds (Sayol et al. 2019), and with findings of increased body-size disparity in bird families with large brains (Sol and Price 2008).

Our results support this idea in finding indications that absolute brain size is related to the rate of evolution of some aspects of beak shape, but with the caveat that relative brain size had little effect. Our models never explained more than a few percent of the variance in beak-shape disparity, but in the cases explaining more than a percent or so of the variance, the best estimates of effects usually indicated that the variance in shape change and thus the disparity would increase substantially. For example, assuming that brain mass evolved as a geometric Brownian motion and had a linear effect on the rate variance of the shape variables leads to the prediction that a clade of birds with twice the brain mass of another would have between 1.5 and 3.5 times the disparity (among-species variance) in many of the beak-shape variables. The recent-evolution model (Model 3) also showed rather consistent positive effects, which indicates that the rapid stationary fluctuations that characterize evolution on short below-species-level time scales are more pronounced in larger-brained birds.

The absence of consistent effects of relative brain size is surprising, but consistent with the idea that it is absolute more than relative brain size that matters for intelligence (e.g., MacLean et al. 2014; and discussed in Striedter 2005). There is a serious caveat, however, in that brain size is related to many other ecological and behavioral factors in birds (e.g., Burish et al. 2004; Shultz and Dunbar 2010; Vincze 2016), and we cannot exclude that some factor other than intelligence mediates the causal effect on the tempo of evolution. We tested the effect of body size on rates and found that these could not fully explain the effect of absolute brain size in Model 2, but that they mirrored the effects of absolute brain size when using the recent-evolution model. It is therefore possible that the elevated microevolutionary rates of large-brained species are mediated through some other correlate of larger bodies.

### Relation to Other Methods

As mentioned in the introduction, there are several methods for studying rates of evolution in phylogenies or fossil time series based on stochastic models. The approach most similar to ours is the likelihood-based method pioneered by O’Meara et al. (2006) and Thomas et al. (2006). Here, Brownian motions with different rate parameters are fitted to different sections (regimes) of a phylogeny. This is similar to our Model 1, but with the predictor variable as a categorical fixed effect mapped onto the phylogeny. This approach is superior to ours when the predictor categories can be mapped reliably onto the phylogeny, because the assumption of a fixed (nonevolving) predictor allows a Gaussian process and hence the application of likelihood methods based on the normal distribution. Our models should be considered an alternative to this only when the predictor is continuous or uncertain such that mapping is unreliable. In this situation a full stochastic model for the joint evolution of both response and predictor variables is preferable. In fact, Revell (2013) has shown that rate estimates from different regimes are biased to be more similar when mapping of regimes is uncertain. Also be aware that the low power we have illustrated is not a particular flaw of our models but apply similarly to all statistical estimators of variance of change.

Another approach to the study of rate involves the estimation of ancestral states on a phylogeny and then computation of rates over ancestor–descendant pairs or contrasts between such estimates. This is often done without consideration of statistical uncertainty and is problematic because estimated ancestral states and derived contrasts are generally uncertain, heteroscedastic, and correlated (Martins and Hansen 1997). As discussed in Hansen (2014), this approach is at best a roundabout way of estimating rate parameters with many pitfalls and no advantages to a direct approach.

A related approach is to correlate disparity at higher taxonomic levels with trait means at those levels, as was done by Wyles et al. (1983) and Sol and Price (2008). See also Weir and Lawson (2016) for a pairwise-contrasts method to study the effects of predictors on evolutionary rates with an application to song complexity in birds.

Our three basic models are far from exhaustive, and there are many conceivable modifications and extensions. Some flexibility can be achieved by scale transformations of the variables, such as use of log scale or not. A more challenging extension would be to multivariate rates. In our example, we decomposed a multivariate character into principal components and analyzed these separately with our univariate models. It is well known, however, that principal-component analysis of comparative data can be misleading because it mixes phylogenetic structure with trait dependence (Uyeda et al. 2015). The fundamental problem with multivariate extensions, however, is that the number of rate parameters will increase, and given the low power of the univariate approaches, any method not involving some form of dimensionality reduction is unlikely to be useful. Extension to more than one predictor variable in the fashion of a multiple regression is also possible but increases mathematical complexity and would likely need to assume independent evolution of the predictors to keep a manageable number of parameters.

The Ornstein–Uhlenbeck process is a common extension to Brownian motion in the stochastic modeling of evolution. Parameters in the Ornstein–Uhlenbeck process are sometimes used to describe rates of evolution (e.g., Martins 1994) or rates of adaptation (Hansen 1997, 2012). These parameters have a less direct interpretation as rates, however, and are perhaps better considered as collectively describing dynamically changing tempo and mode of evolution in relation to selective optima. Nevertheless, one could consider models with either the “rate of adaptation” or the stochastic diffusion of an Ornstein–Uhlenbeck model being influenced by an evolving trait. The moments of such a process would be calculable, but more complicated than in the models we have presented. The regressions would also be nonlinear. Likelihood methods for estimating the influence of mapped “fixed effects” on parameters in Ornstein–Uhlenbeck models have been presented by Beaulieu et al. (2012). Another, simpler, extension would be to allow the predictor variable to follow an Ornstein–Uhlenbeck process in place of the Brownian motion or geometric Brownian motion. Finally, May and Moore (2020) have recently proposed a model similar to ours but with a discrete predictor variable evolving according to a Markov chain. This complements the models in the present article.

The recent-evolution model provides a different perspective on rates of evolution than in most approaches derived from comparative methods or fossil time-series analysis. Instead of studying rates of evolution as change per time over phylogenetic branches or time intervals, this approach focuses on recent evolution after removing the effects of past change. In fact, the different models we have presented resemble two positions from the early days of the phylogenetic comparative-methods literature (reviewed in Martins and Hansen 1996). One position, exemplified by the autoregressive approach of Cheverud et al. (1985), sought to remove the phylogenetic component and study adaptation in the reminder, while another position, exemplified by Lynch’s (1991) mixed model, sought to remove the species-specific component and study adaptation in the phylogenetic component. While the latter position, being implicit in Felsenstein’s (1985) independent-contrasts and related process-based phylogenetic comparative methods, won out for conventional analysis of trait evolution, our analysis illustrates how this can be seen as a distinction between studying evolution at two different time scales, a macroevolutionary, above species-level time scale, and a microevolutionary, below species-level time scale. As argued by Uyeda et al. (2011) and Hansen (2012), evolutionary rates behave differently at micro- and macroevolutionary time scales and likely reflect different evolutionary mechanisms. This justifies the development of distinct models to study rates at different time scales.

The recent-evolution model also resembles comparative methods designed to make inferences about changes at single branches; typically aiming to test if particular changes are unusual as compared to the overall rate of evolution on the phylogeny (e.g., McPeek 1995; Revell 2008; Nunn and Zhu 2014). In fact, our equation 11 could be used to pick out the deviance for any species or subset of species, and test these for conformity to an expected Gaussian distribution with the predicted variance matrix in equation 12. See also Uyeda et al. (2018) for more extensive discussion of the role of singular events in comparative studies.

Our motivation for developing these models was to explore the potential for testing hypotheses about factors influencing rates of evolution or the disparity of a clade. For example, as reviewed in Bolstad et al. (2014), there have been many claims that rates of divergence and among-species variance are related to within-species variance, or to more specific measures of species evolvability. Such claims are usually based on comparing clade variances to estimates of evolvability from one or a few species across different traits, and thus do not fit the framework we have set up. It is, however, desirable to develop direct comparative tests of how measures of evolvability and other microevolutionary parameters influence rates of divergence, and the models presented here illustrate both how this can be done and the limitations in doing so.

## Supplementary Material

Data and supplementary analyses are available from the Dryad Digital Repository: https://doi.org/10.5061/dryad.gb5mkkwq9. Software is available at CRAN: https://CRAN.R-project.org/package=evolvability.

## A.1 Calculation of Moments

In this appendix, we compute the necessary moments for the different models by integration. The general strategy is to use the multivariate Itô formula (e.g., Gard 1988, p. 59) to derive stochastic differential equations for powers of the variables $x$ and $y$ and then integrate over $x$ and $y$ to form differential equations for moments and cross moments. We then solve these differential equations by standard integration over time.

The Model 1 was

$$
\begin{align*}
\begin{aligned}
dy &= \sqrt {a + bx} dW_1, \\
dx &= \sigma dW_2,
\end{aligned}
\end{align*}$$

with initial conditions $y_{0}$ and $x_{0}$. Since $x$ follows standard univariate Brownian motions, its moments at time $t$ are

$$
\begin{align*}
E\left[ {\left( {x-x_0 } \right)^k} \right] = \left\{\begin{array}{ll}
\sigma^kt^{k / 2}(k-1)!!&\mbox{ if }k\mbox{ is even} \\
0&\mbox{ if }k\mbox{ is odd} \\
\end{array}\right.,
\end{align*}$$

where the !! signifies semifactorial. We illustrate the computation of the other moments by an example, E[$y^{2}x$]. The Itô formula yields

$$
\begin{align*}
dy^2x = (a + bx)xdt + 2yx\sqrt {a + bx} dW_1 + \sigma y^2dW_2.
\end{align*}$$

Taking the expectation, and using the fact that the ${\it dW}_{i}$ have mean zero and are independent of previous history, yields

$$
\begin{align*}
dE[y^2x] &= E[(a + bx)x]dt = \left( {aE\left[ x \right] + bE\left[ {x^2}
\right]} \right)dt \\
&= \left( {a + bx_0 } \right)x_0 + b\sigma^2tdt.
\end{align*}$$

Integrating yields

$$
\begin{align*}
E[y^2x] = y_0^2 x_0 + \left( {a + bx_0 } \right)x_0 t + \frac{b\sigma
^2t^2}{2},
\end{align*}$$

and since $E\left[ {y^2} \right] = y_0^2 + \left( {a + bx_0 } \right)t$, we get

$$
\begin{align*}
Cov[y^2,x] = \frac{b\sigma^2t^2}{2}.
\end{align*}$$

The differential equation for a general moment obtained from the Itô formula is

$$
\begin{align*}
\frac{dE\left[ {y^mx^k} \right]}{dt} &= \frac{k\left( {k-1} \right)\sigma
^2}{2}E\left[ {y^mx^{k-2}} \right]\\
&+ \frac{m\left( {m-1}
\right)}{2}E\left[ {\left( {a + bx} \right)y^{m-2}x^k} \right],
\end{align*}$$

which can be solved iteratively in $m$ and $k$. In the following we specifically need the fourth moment of $y$, which is

$$
\begin{align*}
E\left[ {y^4} \right] = y_0^4 + 6\left( {a + bx_0 } \right)y_0^2 t + 3\left(
{a + bx_0 } \right)^2t^2 + b^2\sigma^2t^3,
\end{align*}$$

which yields

$$
\begin{align*}
Var\left[ {y^2} \right] = 4\left( {a + bx_0 } \right)y_0^2 t + 2\left( {a +
bx_0 } \right)^2t^2 + b^2\sigma^2t^3.
\end{align*}$$

Note that this shows that the distribution of $y$ is leptokurtic and not Gaussian.

To obtain the necessary cross-species covariances needed for the GLS regression, we use an approach from Hansen and Martins (1996) based on conditioning on the values of the most recent common ancestor of the two species in question, and assuming that they evolve independently after they split apart. This requires the following conditional expectations:

$$
\begin{align*}
E\left[ {y_i^2 |y_a,x_a } \right] &= y_a^2 + (a + bx_a )t_{ai},\\
E\left[ {x_i |y_a,x_a } \right] &= x_a,
\end{align*}$$

where $y_{a}$ and $x_{a}$ are the values of an ancestor of $i$ and $t_{ai}$ is the time that separates the ancestor $a$ and the species $i$. For an ultrametric tree, $t_{ai}=t_{aj}=t - t_{a}$ with $t_{a}$ the time from root to the most recent common ancestor of $i$ and $j$. From this, we get

$$
\begin{align*}
Cov[y_i^2,y_j^2 ] &= Cov[E[y_i^2 |y_a,x_a ],E[y_j^2 |y_a,x_a ]]\\
&= Var\left[ {y_a^2 + (a + bx_a )(t-t_a )} \right]\\
& = Var\left[ {y_a^2 }
\right] + b^2\left( {t-t_a } \right)^2Var[x_a ]\\
&\quad + 2b\left( {t-t_a }
\right)Cov\left[ {y_a^2,x_a } \right]\\
&= 4\left( {a + bx_0 } \right)y_0^2 t_a + 2\left( {a + bx_0 } \right)^2t_a^2\\
&\quad+ b^2\sigma^2t_a^3 + b^2\left( {t-t_a } \right)^2\sigma^2t_a + b^2\sigma
^2\left( {t-t_a } \right)t_a^2 \\
& = 4\left( {a + bx_0 } \right)y_0^2 t_a + 2\left( {a + bx_0 } \right)^2t_a^2\\
&\quad + b^2\sigma^2t_a \left( {t^2-t_a t + t_a^2 } \right), \\
Cov[y_i^2,x_j ] &= Cov[E[y_i^2 |y_a,x_a ],E[x_j |y_a,x_a ]]\\
&=
Cov\left[ {y_a^2 + (a + bx_a )(t-t_a ),x_a } \right]\\
&= \textstyle{1 \over 2}b\sigma^2t_a^2 + b\left( {t-t_a } \right)\sigma
^2t_a = b\sigma^2\left( {t-\frac{t_a }{2}} \right),\\
Cov\left[ {x_i,x_j } \right] &= Cov\left[ {E[x_i |x_a ],E[x_j |x_a
]} \right] = Var[x_a ] = \sigma^2t_a.
\end{align*}$$

These moments are all we need to construct the necessary variance and covariance matrices for GLS regression. This is detailed in Appendix B.1.

The Model 2 was

$$
\begin{align*}
\begin{aligned}
dy &= \sqrt {a + bx} dW_1, \\
dx &= \textstyle{1 \over 2}\sigma^2xdt + \sigma xdW_2,
\end{aligned}
\end{align*}$$

which implies that $x$ follows a log-normal distribution with moments

$$
\begin{align*}
E\left[ {x^k} \right] = x_0^k e^{\textstyle{1 \over 2}k^2\sigma^2t}.
\end{align*}$$

Using the Itô formula and taking moments yield, the following differential equation for an arbitrary moment of the process:

$$
\begin{align*}
\frac{dE\left[ {y^mx^k} \right]}{dt} = \frac{k^2\sigma^2}{2}E\left[
{y^mx^k} \right] + \frac{m\left( {m-1} \right)}{2}E\left[ {\left( {a + bx}
\right)y^{m-2}x^k} \right],
\end{align*}$$

which can be solved iteratively in $m$. Most pertinently

$$
\begin{align*}
E\left[ {yx^k} \right]& = y_0 x_0^k e^{\textstyle{1 \over 2}k^2\sigma^2t},\\
E\left[ {y^2x^k} \right] &= y_0^2 x_0^k e^{\textstyle{1 \over 2}k^2\sigma
^2t} + ax_0^k te^{\textstyle{1 \over 2}k^2\sigma^2t}\\
&\quad + \frac{2bx_0^{k + 1}
}{\left( {1 + 2k} \right)\sigma^2}\left( {e^{\textstyle{1 \over 2}\left( {k
+ 1} \right)^2\sigma^2t}-e^{\textstyle{1 \over 2}k^2\sigma^2t}}
\right),\\
E\left[ {y^3x^k} \right] &= y_0^3 x_0^k e^{\textstyle{1 \over 2}k^2\sigma
^2t} + 3ay_0 x_0^k te^{\textstyle{1 \over 2}k^2\sigma^2t}\\
&\quad + \frac{6by_0
x_0^{k + 1} }{\left( {1 + 2k} \right)\sigma^2}\left( {e^{\textstyle{1 \over
2}\left( {k + 1} \right)^2\sigma^2t}-e^{\textstyle{1 \over 2}k^2\sigma
^2t}} \right),\\
E\left[ {y^4x^k} \right] &= y_0^4 x_0^k e^{\textstyle{1 \over 2}k^2\sigma
^2t} + 3ax_0^k \left( {2y_0^2 + at} \right)te^{\textstyle{1 \over
2}k^2\sigma^2t}\\
&\quad + \frac{12bx_0^{k {+} 1} \left( {at {+} y_0^2 } \right)}{\left(
{2k {+} 1} \right)\sigma^2}\left( {e^{\textstyle{1 \over 2}\left( {k + 1}
\right)^2\sigma^2t}{-}e^{\textstyle{1 \over 2}k^2\sigma^2t}} \right) \\
&\quad + \frac{6b^2x_0^{k + 2} }{\left( {2k + 3} \right)\left( {2k + 1}
\right)\left( {k + 1} \right)\sigma^4}\\
&\quad \left( \left( {2k + 3}
\right)e^{\textstyle{1 \over 2}k^2\sigma^2t} {+} \left( {2k {+} 1}
\right)e^{\textstyle{1 \over 2}\left( {k^2 {+} 4k {+} 4} \right)\sigma^2t}\right.\\
&\quad \left.-
4\left( {k + 1} \right)e^{\textstyle{1 \over 2}\left( {k + 1}
\right)^2\sigma^2t} \right),
\end{align*}$$

which shows that Cov[$y, x^{k}] = 0$ and Cov[$y^{3}, x^{k}] = 0$ for all $k$. The even powers of $y$ covary with $x$, however, and this justifies the regression of the square of $y$ on $x$. Using these moments we can compute the cross-species covariances with the same method as for Model 1. To do this, we specifically need

$$
\begin{align*}
E\left[ {y_i |y_a,x_a } \right] &= y_a,\\
E\left[ {y_i^2 |y_a,x_a } \right] &= y_a^2 + at_{ai} + \frac{2b}{\sigma
^2}\left( {e^{\textstyle{{\sigma^2} \over 2}t_{ai} }-1} \right)x_a
\end{align*}$$

This yields

$$
\begin{align*}
Cov[y_i^2,y_j^2 ]& = Cov[E[y_i^2 |y_a,x_a ],E[y_j^2 |y_a,x_a ]] \\
& = Var\left[ {y_a^2 + a\left( {t-t_a } \right) + \frac{2b}{\sigma ^2}\left( {e^{\textstyle{{\sigma^2} \over 2}\left( {t-t_a } \right)}-1} \right)x_a } \right] \\
& = Var\left[ {y_a^2 } \right] + \frac{4b^2}{\sigma^4}\left( {e^{\textstyle{{\sigma^2} \over 2}\left( {t-t_a } \right)}-1}
\right)^2Var[x_a]\\
& + \frac{4b}{\sigma^2}\left( {e^{\textstyle{{\sigma^2} \over 2}\left( {t-t_a } \right)}-1} \right)Cov\left[ {y_a^2,x_a}\right],
\end{align*}$$

$$
\begin{align*}
Cov[y_i^2,x_j ] &= Cov[E[y_i^2 |y_a,x_a ],E[x_j |y_a,x_a ]] \\
& = Cov\left[ {y_a^2 + at_{ai} + \frac{2b}{\sigma^2}\left( {e^{\textstyle{{\sigma^2} \over 2}t_{ai} }-1} \right)x_a,e^{\textstyle{1 \over 2}\sigma^2t_{aj} }x_a } \right] \\
& = e^{\textstyle{1 \over 2}\sigma^2\left( {t-t_a } \right)}Cov[y_a^2,x_a ]\\
&\quad + \frac{2b}{\sigma^2}\left( {e^{\sigma^2\left( {t-t_a } \right)}-e^{\textstyle{1 \over 2}\sigma^2\left( {t-t_a } \right)}}
\right)Var[x_a],\\
Cov\left[ {x_i,x_j } \right] &= Cov\left[ {E[x_i |x_a ],E[x_j |x_a ]} \right] = e^{\sigma^2\left( {t-t_a } \right)}Var[x_a ].
\end{align*}$$

The specific moments needed to solve this are

$$
\begin{align*}
Var\left[ {y^2} \right] &= 4ay_0^2 t + 2a^2t^2 + \frac{8bx_0 \left( {at +
y_0^2 } \right)}{\sigma^2}\left( {e^{\textstyle{1 \over 2}\sigma^2t}-1}
\right)\\
&\quad + \frac{2b^2x_0^2 }{\sigma^4}\left( {e^{\sigma^2t}-1}
\right)^2,\\
Cov\left[ {y^2,x} \right] &= \frac{2bx_0^2 e^{\textstyle{1 \over 2}\sigma
^2t}}{3\sigma^2}\left( {2 + e^{\textstyle{3 \over 2}\sigma^2t}-
3e^{\textstyle{1 \over 2}\sigma^2t}} \right),\\
Var\left[ x \right] &= x_0^2 e^{\sigma^2t}\left( {e^{\sigma^2t}-1}
\right),
\end{align*}$$

which yields

$$
\begin{align*}
Cov[y_i^2,y_j^2 ]& = 4ay_0^2 t_a + 2a^2t_a^2 + \frac{8bx_0 }{\sigma
^2}\left( {y_0^2 + at_a } \right)\left( {e^{\textstyle{1 \over 2}\sigma
^2t_a }-1} \right) \\
&\quad + \frac{2b^2x_0^2 }{\sigma^4}\left( \left( {e^{\sigma^2t}-1} \right)^2
- \left( {e^{\sigma^2t}-e^{\sigma^2t_a }} \right)^2\right.\\
&\quad \left.+ \frac{8}{3}\left(
{1-e^{\textstyle{3 \over 2}\sigma^2t_a}} \right)\left({e^{\textstyle{1
\over 2}\sigma^2t}-e^{\textstyle{1 \over 2}\sigma^2t_a}}\right)
\right),\\
Cov[y_i^2,x_j ] &= \frac{2bx_0^2 }{\sigma^2}e^{\sigma^2t}\left( {e^{\sigma
^2t_a }{-}1{-}\frac{2}{3}\left( {e^{\textstyle{1 \over 2}\sigma^2\left(
{3t_a{-}t} \right)}{-}e^{-\textstyle{1 \over 2}\sigma^2t}} \right)}
\right),\\
Cov\left[ {x_i,x_j } \right]& = x_0^2 e^{\sigma^2t}\left( {e^{\sigma^2t_a
}-1} \right),
\end{align*}$$

which is what is needed to set up the GLS regression in Appendix B.1.

## B.1 Constructing the Regression Matrices

In this appendix, we derive the variance and covariance matrices for the GLS regression of squared y-values on the predictor x-variables. Let ** y**$^{2}$ denote the column vector of species squared y-values. Proposition 2.22 in Eaton (1983) then gives

$$
\begin{align*}
\widehat{{{\bf y}}^2} = E\left[ {{{\bf y}}^2} \right] + Cov[{{\bf y}}^2, {{\bf x}}]Var[ {{\bf x}}]^{-1}\left( { {{\bf x}}- E[{{\bf x}}]} \right),
\end{align*}$$

as the best linear predictor of the ** y**$^{2}$ vector from the ** x** vector in the least-squares sense. This has residual variance matrix

$$
\begin{align*}
{{Var}}\left[ {{\bf r}} \right] &= {{Var}}\left[ {{{\bf y}}^{{\bf 2}}-{{\hat{\bf y}}^{{\bf 2}}}
} \right]\\
&= {{Var}}\left[ {{{\bf y}}^{{\bf 2}}}
\right]-{Cov {\bf [y}}^{{\bf 2}}{{\bf,x]}Var}[{{\bf x}}]^{- 1}{Cov {\bf [y}}^{{\bf 2}}{{\bf,x]}}^T,
\end{align*}$$

where ${Cov {\bf [y}}^{{\bf 2}}{{\bf,x]}}$ is the matrix with elements $Cov[y_i^2,x_j ]$, ${{Var}}[{{\bf x}}]$ is the matrix with elements $Cov\left[ {x_i,x_j } \right]$, and ${Var {\bf [y}}^{{\bf 2}}{{\bf ]}}$ is the matrix with elements $Cov[y_i^2,y_j^2 ]$.

For Model 1, the moments derived in Appendix A.1 then gives:

$$
\begin{align*}
\widehat{{{\bf y}}^2} &= \left( {a + bx_0 +
y_0^2 } \right){{\bf 1}} + b\left( {{{\bf I}}-\textstyle{1 \over
2}\left( {{{\bf T}} \circ {{\bf T}}} \right){{\bf T}}^{-1}}
\right){{\bf x}},\\
Var\left[ {{\bf r}} \right] &= 4\left( {a + bx_0 } \right)y_0^2 {{\bf
T}} + 2\left( {a + bx_0 } \right)^2{{\bf T}} \circ {{\bf T}}\\
&\quad +
b^2\sigma^2\left( {{{\bf T}} \circ {{\bf T}} \circ {{\bf T}}-
\textstyle{1 \over 4}\left( {{{\bf T}} \circ {{\bf T}}} \right){{\bf T}}^{-1}\left( {{{\bf T}} \circ {{\bf T}}} \right)} \right),
\end{align*}$$

where ${\bf T} = [t_{a}]$ is the matrix of shared branch lengths in units of total tree length, ** I** is the identity matrix, ** 1** is a column vector of ones, and $y_{0}$ and $x_{0}$ are the initial values of $y$ and $x$ at the root of the tree.

Centering the variables around estimates of the root values would in principle justify setting $y_{0}=x_{0} = 0$, but the estimation error in the root values can be substantial if there are only a few branches near the root of the phylogeny. To account for this we assume that the estimates of the root values are unbiased and uncorrelated with variance $v_{y}$ for $y_{0}$ and $v_{x}$ for $x_{0}$. Furthermore, we have to account for the fact that the error in estimating $y_{0}$ is correlated with $y^{2}$. To do this, assume the y-variable has evolved as a Brownian motion, so that the y-vector is distributed as ** y**$\sim $ N($y_{0}$**1**, $a$** T**). Let

$$
\begin{align*}
m = \frac{{{\bf 1}}^T{{\bf T}}^{-1}{{\bf y}}}{{{\bf 1}}^T{{\bf T}}^{-1}{{\bf 1}}}
\end{align*}$$

be our (unbiased) estimator of $y_{0}$. Then for species $i$,

$$
\begin{align*}
&E\left[ {\left( {y_i-y_0-\left( {m-y_0 } \right)} \right)^2} \right] =
E\left[ {\left( {y_i-y_0 } \right)^2} \right] + Var\left[ m \right]\\
&-
2Cov[y_i,m] = E\left[ {\left( {y_i-y_0 } \right)^2} \right]-v_y,
\end{align*}$$

which follows because $Cov[y_i,m] = Var\left[ {y_i } \right] / {{\bf 1}}^T{{\bf T}}^{-1}{{\bf 1}} = v_y $. Hence, to get an unbiased estimate of $a$ from the intercept in the regression we subtract $v_{y}$. To correct the residual variance we take the expectation over $y_{0}$ and $x_{0}$. This yields

$$
\begin{align*}
\widehat{{{\bf y}}^{{\bf 2}}} &= \left( {a-v_y }
\right){{\bf 1}} + b\left( {{{\bf I}}-\textstyle{1 \over 2}\left(
{{{\bf T}} \circ {{\bf T}}} \right){{\bf T}}^{-1}} \right){{\bf
x}},\\
Var\left[ {{\bf r}} \right] &= 4av_y {{\bf T}} + 2\left( {a^2 +
b^2v_x } \right){{\bf T}} \circ {{\bf T}}\\
&\quad + b^2\sigma^2\left( {{{\bf T}} \circ {{\bf T}} \circ {{\bf T}}-\textstyle{1 \over
4}\left( {{{\bf T}} \circ {{\bf T}}} \right){{\bf T}}^{-
1}\left( {{{\bf T}} \circ {{\bf T}}} \right)} \right),
\end{align*}$$

and thus equations 3 and 4 in the main text.

For Model 2, the moments derived in appendix A give the following matrix form of the regression

$$
\begin{align*}
\widehat{{{\bf y}}^2} \quad &= \left( {a + \frac{2b}{\sigma^2}\left( {e^{\frac{1}{2}\sigma^2}-1} \right)x_0 + y_0^2 } \right){\bf 1}\\
&\quad + \frac{2b}{\sigma^2}\left( {{{\bf I}}-
\frac{2}{3}e^{-\frac{1}{2}\sigma^2}\left[ {e^{\frac{3}{2}\sigma^2t_a }-
1} \right]\left[ {e^{\sigma^2t_a }-1} \right]^{-1}} \right)\\
&\left( {{{\bf x}}-E[{{\bf x}}]} \right),
\end{align*}$$

$$
\begin{align*}
Var\left[ {{\bf r}} \right] &= 4ay_0^2 {{\bf T}} + \frac{8by_0^2 x_0
}{\sigma^2}\left[ {e^{\textstyle{1 \over 2}\sigma^2t_a }-1} \right] +
2a^2{{\bf T}} \circ {{\bf T}}\\
&\quad + \frac{8abx_0 }{\sigma^2}{{\bf
T}} \circ \left[ {e^{\textstyle{1 \over 2}\sigma^2t_a }-1} \right] \\
&\quad + \frac{2b^2x_0^2 }{\sigma^4}\left( \frac{8}{3}\left[ {e^{2\sigma^2t_a }
- e^{\textstyle{1 \over 2}\sigma^2t_a }} \right]-\left[ {e^{2\sigma^2t_a
}-1} \right]\right.\\
&\left.\quad -\frac{8}{9}\left[ {e^{\textstyle{3 \over 2}\sigma^2t_a }-
1} \right]\left[ {e^{\sigma^2t_a }-1} \right]^{-1}\left[
{e^{\textstyle{3 \over 2}\sigma^2t_a }-1} \right] \right),
\end{align*}$$

where the notation [f($t_{a}$)] means a matrix in which the ij’th element is f($t_{a}$) with $t_{a}$ being the shared branch length between species $i$ and $j$. In this notation ${\bf T} = [t_{a}]$. As for Model 1, we center the y-variable on its estimated root value, but for the x-variable we standardize by dividing with the predicted root value. To model the error in this root value, we assume that ln($x_{0}$) is normally distributed with zero mean and variance $v_{lnx}$. Then

$$
\begin{align*}
E\left[ {x_0} \right]& = e^{\textstyle{1 \over 2}v_{\ln x} },\\
E\left[ {x_0^2 } \right]& = e^{2v_{\ln x}},\\
E\left[ {y_0^2 x_0} \right]& = v_y e^{\textstyle{1 \over 2}v_{\ln x} }.
\end{align*}$$

Using these expectations, we get

$$\begin{align*}
\widehat{{{\bf y}}^2}& = \left( {a + \frac{2be^{\frac{1}{2}v_{lnx}
}}{\sigma^2}\left( {e^{\frac{1}{2}\sigma^2}-1} \right)-v_y } \right){\bf 1}\\
&\quad + \frac{2b}{\sigma^2}\left( {{{\bf I}} - \frac{2}{3}e^{-\frac{1}{2}\sigma^2}\left[
{e^{\frac{3}{2}\sigma^2t_a }-1} \right]\left[ {e^{\sigma^2t_a }-1}
\right]^{-1}} \right)\\
&\quad \left( {{{\bf x}}-e^{\frac{\sigma^2}{2} - \frac{v_{lnx} }{2}} {\bf 1}} \right),\end{align*}$$

$$
\begin{align*}
Var\left[ {{\bf r}} \right] &= 4av_y {{\bf T}} + \frac{8bv_y
e^{\textstyle{1 \over 2}v_{\ln x} }}{\sigma^2}\left[ {e^{\textstyle{1 \over
2}\sigma^2t_a }-1} \right] + 2a^2{{\bf T}} \circ {{\bf T}} \\
&\quad +
\frac{8abe^{\textstyle{1 \over 2}v_{\ln x} }}{\sigma^2}{{\bf T}} \circ
\left[ {e^{\textstyle{1 \over 2}\sigma^2t_a }-1} \right] \\
&\quad + \frac{2b^2e^{2v_{\ln x} }}{\sigma^4}\left( \frac{8}{3}\left[
{e^{2\sigma^2t_a }-e^{\textstyle{1 \over 2}\sigma^2t_a }} \right]-
\left[ {e^{2\sigma^2t_a }-1} \right]\right.\\
&\quad \left.-\frac{8}{9}\left[ {e^{\textstyle{3
\over 2}\sigma^2t_a }-1} \right]\left[ {e^{\sigma^2t_a }-1} \right]^{
- 1}\left[ {e^{\textstyle{3 \over 2}\sigma^2t_a }-1} \right] \right),
\end{align*}$$

assuming that $y$ is centered and $x$ standardized on their predicted root values. This yields equations 7 and 8 in the main text. Note that the second term in the intercept of regression depends on the regression slope. This caused numerical problems in our estimation procedure, which we solved by treating the whole intercept (except $v_{y}$) as a constant, $A$, and the subtracting the second term at the end to estimate $a$.

For Model 3, let $\left( { {\boldsymbol{y}} - \hat{{\boldsymbol{y}}}} \right) \circ \left( {{\boldsymbol{y}} - \hat{{\boldsymbol{y}}}} \right)$ be the column vector of squared deviations from the prediction as given in equation 11. The linear regression of the squared deviations vector on the vector ** x** is

$$
\begin{align*}
&\left( {{\boldsymbol{y}} - \hat{{\boldsymbol{y}}}} \right) \circ \left( {{\boldsymbol{y}} - \hat{{\boldsymbol{y}}}} \right) = E\left[{\left( {{\boldsymbol{y}} - \hat{{\boldsymbol{y}}}} \right) \circ \left( {{\boldsymbol{y}} - \hat{{\boldsymbol{y}}}} \right)} \right]\\
&\quad + Cov\left[ {\left( {{\boldsymbol{y}} - \hat{{\boldsymbol{y}}}} \right) \circ \left( {{\boldsymbol{y}} - \hat{{\boldsymbol{y}}}} \right),{{\bf
x}}} \right]Var\left[ {{\bf x}} \right]^{ - 1}\left( {{{\bf x}} -
{\bar{\bf x}}} \right) + {\bf r},
\end{align*}$$

where ** r** is a vector of residuals. To compute this we use

$$
\begin{align*}
&E\left[ {\left( {{\boldsymbol{y}} - \hat{{\boldsymbol{y}}}} \right) \circ \left( {{\boldsymbol{y}} - \hat{{\boldsymbol{y}}}} \right)}\right]\\
&\quad = diag\left[ {Var\left[ {\left( {{\boldsymbol{y}} - \hat{{\boldsymbol{y}}}} \right)} \right]} \right]\\
&\quad = diag\left[ {Var\left[ {d\left( {{{\bf V}}^{ - 1}} \right)^{ -1}{{\bf V}}^{ - 1}\left( {{{\bf y}} - \hat{{\boldsymbol{y}}}} \right)} \right]} \right]\\
&\quad = diag\left[ {d\left( {{{\bf V}}^{ - 1}} \right)^{ - 1}{{\bf V}}^{ - 1}Var\left[ {\left( {{{\bf y}} - \hat{{\boldsymbol{y}}}} \right)} \right]{{\bf V}}^{ - 1}d\left( {{{\bf V}}^{ - 1}} \right)^{ - 1}} \right]\\
&\quad = diag\left[ {d\left( {{{\bf V}}^{ - 1}} \right)^{ - 1}{{\bf V}}^{ - 1}\left( {{{\bf V}}_{macro} {+} {{\bf V}}_{micro} } \right){{\bf V}}^{ - 1}d\left( {{{\bf V}}^{ - 1}} \right)^{ - 1}} \right]\\
&\quad = diag\left[ {d\left( {{{\bf V}}^{ - 1}} \right)^{ - 1}{{\bf V}}^{ - 1}{{\bf V}}_{macro} {{\bf V}}^{ - 1}d\left( {{{\bf V}}^{ - 1}} \right)^{ - 1}} \right]\\
&\qquad + diag\left[ d\left( {{{\bf V}}^{ - 1}} \right)^{ - 1}{{\bf V}}^{ - 1}\left( a{{\bf I}}\right.\right.\\
&\qquad\quad \left.\left.{+} E\left[ {diag\left( {{{\bf x}} {-} {\bar{\bf {x}}}} \right)} \right] \right){{\bf V}}^{ - 1}d\left( {{{\bf V}}^{ - 1}} \right)^{ - 1} \right]
\end{align*}$$

and

$$
\begin{align*}
&Cov\left[ {\left( {{\boldsymbol{y}} - \hat{{\boldsymbol{y}}}} \right) \circ \left( {{\boldsymbol{y}} - \hat{{\boldsymbol{y}}}} \right),{{\bf x}}} \right]\\
&= Cov\left[ {d\left( {{{\bf V}}^{ - 1}} \right)^{ - 1}{{\bf V}}^{ - 1}\left( {{{\bf y}} - {\hat{{\boldsymbol{y}}}}} \right) \circ d\left( {{{\bf V}}^{ - 1}} \right)^{ - 1}{{\bf V}}^{ - 1}\left( {{{\bf y}} - \bar{y}} \right),{{\bf x}}} \right]\\
&= b\left( {d\left( {{{\bf V}}^{ - 1}} \right)^{ - 1}{{\bf V}}^{ - 1} \circ d\left( {{{\bf V}}^{ - 1}} \right)^{ - 1}{{\bf V}}^{ - 1}} \right)Var[{{\bf x}}],
\end{align*}$$

which can be verified by elementwise comparison using the assumption that Cov[$y_{r}y_{k}$, $x_{i}] =$ Cov[E[$y_{r}y_{k}|{\bf x}],x_{i}] =$$b$Cov[$x_{i}, x_{j}$] if $r=k=j$, and zero if $r \ne k$. Note that we have treated predictor as fixed, and ** V** as a constant in the macroevolutionary prediction. This yields equations 14 and 15 in the main text.

Observation, that is measurement, error in the species means can also be added to the residual variance matrices. Let ** V**$_{\bf my}$ and ** V**$_{\bf mx}$ be the measurement variance matrices for the response (i.e., the y-squared vector) and predictor (i.e. the x-vector). These matrices will typically be diagonal, but in principle measurement covariances can also be included. The terms added to the residual variance will be then be

$$
\begin{align*}
{{\bf V}}_{my} + b^2\left( {{{\bf I}} - \textstyle{1 \over 2}\left(
{{{\bf T}} \circ {{\bf T}}} \right){{\bf T}}^{ - 1}} \right){
{\bf V}}_{mx} \left( {{{\bf I}} - \textstyle{1 \over 2}\left( {{{\bf
T}} \circ {{\bf T}}} \right){{\bf T}}^{ - 1}} \right)^T
\end{align*}$$

for Model 1 and

$$
\begin{align*}
&{{\bf V}}_{my} + \frac{4b^2}{\sigma ^4}\left( {{{\bf I}} -
\textstyle{2 \over 3}e^{ - \textstyle{1 \over 2}\sigma ^2}\left[
{e^{\textstyle{3 \over 2}\sigma ^2t_a } - 1} \right]\left[ {e^{\sigma ^2t_a
} - 1} \right]^{ - 1}} \right)\\
&{{\bf V}}_{mx} \left( {{{\bf I}} -
\textstyle{2 \over 3}e^{ - \textstyle{1 \over 2}\sigma ^2}\left[
{e^{\textstyle{3 \over 2}\sigma ^2t_a } - 1} \right]\left[ {e^{\sigma ^2t_a
} - 1} \right]^{ - 1}} \right)^T
\end{align*}$$

for Model 2. For Model 3, measurement variance can be added as described in the main text.

Observation error in the predictor variable may also cause attenuation, a downward bias in the estimated regression slope. Attenuation is not corrected by including observation error in the residual variance. Expressions for attenuation with correlated data are given in Hansen and Bartoszek (2012). Applying this to our situation yields the attenuation factor

$$
\begin{align*}
K = 1 - \frac{\begin{array}{c}\left( {{{\bf x}} - {{\bf 1}}} \right)^T\left( {{
Cov {\bf [y}}^{{\bf 2}}{{\bf,x]}Var}[{{\bf x}}]^{ - 1}}
\right)^T\\
{Cov {\bf [y}}^{{\bf 2}}{{\bf,x]}Var}[{{\bf x}}]^{
- 1}{{Var}}[{{\bf r}}]^{ - 1}{Cov {\bf [y}}^{{\bf 2}}{
{\bf,x]}Var}[{{\bf x}}]^{ - 1}\\
{{\bf V}}_{mx}{{Var}}[{
{\bf x}}]^{ - 1}\left( {{{\bf x}} - {{\bf 1}}} \right)\end{array}}
{\begin{array}{c}\left( {{
{\bf x}} - {{\bf 1}}} \right)^T
\left( {{Cov {\bf [y}}^{{\bf
2}}{{\bf,x]}Var}[{{\bf x}}]^{ - 1}} \right)^T\\
{{Var}}[{
{\bf r}}]^{ - 1}{Cov {\bf [y}}^{{\bf 2}}{{\bf,x]}Var}[{{\bf
x}}]^{ - 1}\left( {{{\bf x}} - {{\bf 1}}} \right)\end{array}}.
\end{align*}$$

Dividing the estimated slope with $K$ would give an approximately unbiased estimate of the true slope.
